# Exploring the role of COVID-19 pandemic-related changes in social interactions on preschoolers' emotion labeling

**DOI:** 10.3389/fpsyg.2022.942535

**Published:** 2022-09-28

**Authors:** Stephanie Wermelinger, Lea Moersdorf, Simona Ammann, Moritz M. Daum

**Affiliations:** Department of Psychology, University of Zurich, Zurich, Switzerland

**Keywords:** COVID-19, facial masks, emotion recognition, children, gaze behavior

## Abstract

During the COVID-19 pandemic people were increasingly obliged to wear facial masks and to reduce the number of people they met in person. In this study, we asked how these changes in social interactions are associated with young children's emotional development, specifically their emotion recognition *via* the labeling of emotions. Preschoolers labeled emotional facial expressions of adults (Adult Faces Task) and children (Child Faces Task) in fully visible faces. In addition, we assessed children's COVID-19-related experiences (i.e., time spent with people wearing masks, number of contacts without masks) and recorded children's gaze behavior during emotion labeling. We compared different samples of preschoolers (4.00–5.75 years): The data for the *no-COVID-19-experience sample* were taken from studies conducted before the pandemic (Adult Faces Task: *N* = 40; Child Faces Task: *N* = 30). The data for the *with-COVID-19-experience* sample (*N* = 99) were collected during the COVID-19 pandemic in Switzerland between June and November 2021. The results did not indicate differences in children's labeling behavior between the two samples except for fearful adult faces. Children with COVID-19-experience more often labeled fearful faces correctly compared to children with no COVID-19 experience. Furthermore, we found no relations between children's labeling behavior, their individual COVID-19-related experiences, and their gaze behavior. These results suggest that, even though the children had experienced differences in the amount and variability of facial input due to the pandemic, they still received enough input from visible faces to be able to recognize and label different emotions.

## 1. Introduction

Since the beginnings of the COVID-19 pandemic in early 2020, this historic event has dramatically changed people's social life: People had to communicate *via* video calls, keep distance when encountering each other, reduce the number of people they meet in person, and stay at home whenever possible. Among other factors, these changes have led to decreased wellbeing and higher stress and depression levels (Lannen et al., 2020; Russell et al., 2020; Cerniglia et al., 2021). Additionally, the World Health Organization (WHO) recommended wearing facial masks as part of the strategy to slow down the spread in June 2020. Not only adults but also children had to adapt to these different ways of interacting with others. In this study, we explored whether and how differences in social interactions (with a focus on seeing fewer people without masks and an increased amount of facial masks) relate to children's facial emotion recognition, more specifically, emotion labeling.

Facial emotion recognition is particularly interesting in this context because its development is influenced by the input children receive (e.g., Pollak et al., 2009). This input is dependent on the context in which children live and is likely to have changed in the pandemic. For instance, during the pandemic, children saw fewer people and the same faces more often (i.e., their parents). The variability in their facial input may therefore have been reduced. Furthermore, facial masks cover the mouth and nose region, concealing facial features important for recognizing emotions (Gori et al., 2021; Schneider et al., 2021). To our knowledge, there are only few studies on preschoolers' emotion recognition (Gori et al., 2021; Schneider et al., 2021), which investigated the recognition of emotions like joy, anger, fear, sadness, and neutrality in adult faces with and without facial masks. However, these studies only compared children's emotion recognition in faces with versus without mask in a limited number of emotions, ignoring the role of pandemic experiences. Consequently, they cannot speak to broader effects of the pandemic and whether such effects would transfer to emotion recognition in faces without masks. In this study, we investigated children's emotion recognition *via* their ability to label emotions depicted in static faces. We sought to extend previous findings in multiple ways. First, we aimed to gain broader insights into pandemic effects, including potential effects of changed social interactions. For this, we compared children with COVID-19 experience to children without COVID-19 experience regarding their emotion labeling in fully visible faces. Second, in the with-COVID-19-experience sample, we assessed two variables that might be related to children's emotion labeling: The time children spent with others wearing facial masks and the number of contacts without facial masks. Third, we aimed to provide a more fine-grained investigation of children's emotion labeling. Therefore, we included a larger number of different emotions, depicted by children and adults. Finally, we wanted to explore potential associations between children's gaze behavior and their emotion labeling. For this, we assessed children's gaze behavior by using eye tracking. Taken together, this study aimed at understanding children's facial emotion recognition *via* labeling in fully visible faces and how it is associated with pandemic-related changes, such as changes in social interactions and differences in facial input children receive. This provides first insights into how children's emotion recognition might be influenced beyond situations in which masks are worn and therefore beyond the pandemic.

### 1.1. The relevance of facial emotion recognition

Facial expressions are one of the primary social signals, allowing people to draw conclusions about their interaction partners' feelings, intentions, and beliefs (Baron-Cohen, 1995; Ekman, 2007). Moreover, Ekman and Friesen (1971) provided evidence that the recognition of the facial expressions of basic emotions (i.e., surprise, fear, disgust, anger, happiness, and sadness) is universal, meaning that basic emotions are similarly expressed in the face and decoded across cultures worldwide (Ekman and Friesen, 1971).

The ability to recognize and respond to other people's expressive behavior constitutes a fundamental base for social and emotional development (Caron et al., 1982). Furthermore, facial emotion recognition in particular is associated with children's cognitive and linguistic development (Blair, 2002), including social skills and teacher-rated academic competence (Izard et al., 2001; Denham et al., 2015). The likelihood of showing psychopathology (Southam-Gerow and Kendall, 2002) or externalizing and internalizing problems (Trentacosta and Fine, 2010) rises with the difficulty to understand emotions shown in faces. Moreover, despite an overall improvement in facial emotion recognition with age, early individual differences persist across the lifespan (Pons and Harris, 2005).

### 1.2. The measurement and development of facial emotion recognition

The ability to read others' emotions through facial expressions develops across childhood (Herba et al., 2006). However, it is difficult to draw a consistent picture of this development because assessment methods differ with children's age. In the current study, we focus on emotion labeling, the most widely used method within our age group of interest (e.g., Gagnon et al., 2014; Guarnera et al., 2017). In these tasks, children see an emotional facial expression and either freely label it or choose from a certain set of labels. Based on these tasks, it has been proposed that children initially evaluate emotions valence-based (Widen and Russell, 2008; Widen, 2013; Martins et al., 2016) and gradually change to a category-based recognition throughout development (Widen and Russell, 2008). Furthermore, children seem to acquire emotion labels in a certain developmental order (Widen and Russell, 2003, 2008). For instance, between 3 and 4 years, children correctly label happiness, anger, and sadness. Whereas, they show the greatest accuracy for happy expressions, anger is used for both, angry and disgusted faces, and sadness for sad and fearful faces (Widen and Russell, 2003). Later, at the age of 5 years, children label happy, angry, sad, surprised, and fearful faces correctly.

Furthermore, children's early experiences play a critical role in the development of face and probably also emotion recognition (e.g., Taylor-Colls and Pasco Fearon, 2015). For instance, early experience with specific types of faces leads to lasting advantages in processing these faces (Kelly et al., 2007; Park et al., 2009). In a similar vein, early experiences with certain facial emotions may also explain why children recognize these emotions better, as supported by findings on children, who were exposed to high levels of parental anger and physical threat. Not only were these children able to recognize anger with fewer facial cues than children not being exposed to these stressors, but also their parents' reported degree of anger/hostility was related to how fast the children recognized anger (Pollak et al., 2009). Similarly, maternal depression in combination with negative parenting (i.e., parental hostility or high expression of frustration) is associated with reduced emotion recognition in preschoolers (Kujawa et al., 2014).

In sum, experience seems to shape children's emotion recognition. This might be particularly important when children interact in a social world that has changed due to the pandemic: They increasingly interact with adults wearing facial masks, with fewer adults without facial masks, and might encounter certain emotions in different frequencies than before the pandemic (e.g., more negative emotions based on increased stress, fear, and depressive states). These experiences may influence their ability to recognize emotions in others' faces, even in situations where their faces are not covered with a facial mask.

### 1.3. The role of certain facial features for emotion recognition

Adults process faces holistically (Tanaka and Sengco, 1997). For children, the picture is less clear. Carey and Diamond (1994) suggested that already 4- to 6-year-old process faces holistically like adults. However, Schwarzer (2002) provided evidence that 2- to 5-year-old rely more on individual facial features and less on holistic processing when categorizing faces. Which role do facial features play in emotion recognition? Like adults, children recognize different emotions from certain facial features. For instance, when being asked to recognize facial emotions, children until 9 years preferably process the eye area, and occluding other features of the face (such as the mouth) does not impair their emotion recognition (Roberson et al., 2012). In contrast, Guarnera et al. (2017) provided no evidence for differences in looks to the eyes and mouth for emotion recognition in 6- to 7-year-old children. Furthermore, Kestenbaum (1992) found that fear, surprise, and anger were better recognized from the eyes than from the mouth, while happiness was better recognized from the mouth by 5- to 7-year-old. In another study with 5-year-old, fear was best recognized from the upper face half and surprise from the lower face half as well as from the complete face (Gagnon et al., 2014). Guarnera et al. (2015) found that 6- to 7-year-old children generally recognize emotions better when pictures represent the whole face, except for sadness, which is best recognized from the eyes, whereas anger can be identified from the eyes as well as from the whole face.

Although the existing research regarding the processing of specific emotions and the importance of different facial features is not always consistent, most studies indicate an emotion-specific processing of facial expressions. This might be particularly important when investigating the effect of facial masks on the processing of emotions because masks cover only the lower part of the face while the eyes remain visible. Previous studies on the influence of facial masks on emotion recognition showed that emotional expressions are correctly recognized in faces that were covered with masks (Calbi et al., 2021), but that 7- to 13-year-old children were more accurate when faces were fully visible (Ruba and Pollak, 2020). Carbon (2020) suggests that emotion recognition in faces wearing masks is reduced with the exception of fearful and neutral expressions. However, so far little is known about the long-term effects of seeing people wearing masks on children's emotion recognition in fully visible faces.

In sum, previous studies suggest that the occlusion of faces with facial masks has an influence on children's emotion recognition (Carbon, 2020; Ruba and Pollak, 2020) and that this influence might depend on the emotion expressed (Kestenbaum, 1992; Gagnon et al., 2014). Furthermore, children's early experiences seem to alter how they perceive others' facial emotions. Therefore, long-term exposure to people wearing facial masks, reduced number of contacts without facial masks, and changed frequencies of observing certain emotions (*COVID-19-related experiences*) may provide children with fewer and different learning opportunities with emotional expressions in fully visible faces. This could lead to altered emotion recognition, even when there is no facial mask present in the processed face.

### 1.4. The present study

With the present study, we aimed to answer the following research question: Do preschoolers of a sample assessed during the COVID-19-related changes in social interactions show a different emotion recognition (assessed *via* emotion labeling) in fully visible faces than preschoolers from another sample assessed before the COVID-19-related changes?

We deem this question particularly relevant because it investigates one of the major concerns parents and the society repeatedly expressed, namely whether the changes in social interactions might have long-term consequences on emotion recognition, that is, when children process fully visible faces. To address this research question, we applied a cross-sectional research design using the COVID-19 pandemic-related changes in social interactions as a natural intervention. We compared data of preschoolers' emotion labeling from two studies published in 2010 by Widen and Russel and in 2020 by Streubel and colleagues (*no-COVID-19-experience samples*) to data of a new sample of children who had substantial experience with COVID-related changes (*with-COVID-19-experience sample*), which was recruited for this study. We measured children's emotion recognition *via* emotion labeling in fully visible faces in two tasks. In one task, children freely labeled emotional facial expressions of adults (*Adult Faces Task*; task adapted from Widen and Russell, 2010). In a second task, the children did the same with emotional facial expressions of children (*Child Faces Task*; task adapted from Streubel et al., 2020). We chose adult and child faces to make the stimuli more ecologically valid and to mirror previous research which also explored both types of stimuli (Boyatzis et al., 1993; Gagnon et al., 2014). Furthermore, in the with-COVID-19-experience sample, we assessed a subset of children's COVID-19-related experiences (i.e., time seeing people wearing facial masks, number of contacts without facial masks) with a parental questionnaire (self-developed items) and recorded children's gaze behavior *via* eye tracking to explore potential associations with children's emotion labeling.

As the target group we chose children of four to five years. By this age, children can already recognize and name most of the basic emotions, whereas their emotion categories are still developing (Widen and Russell, 2008). As a result, children at this age show some variance in terms of their ability to label different emotions (Widen and Russell, 2003, 2008).

We formulated two hypotheses: First, even though some studies argue that children preferably focus on the eye region (Roberson et al., 2012) and masks would therefore not influence their emotion recognition, the literature is inconsistent with respect to the specific information children use to process facial emotions. For instance, studies show that in general children process faces in a holistic way (Carey and Diamond, 1994) and therefore are better in recognizing emotions shown in the whole face (Guarnera et al., 2015). Furthermore, some studies showed that emotion recognition is more accurate when faces are fully visible compared to faces with masks (Carbon, 2020; Ruba and Pollak, 2020). As a result, children might show less accurate emotion labeling after the COVID-19-related changes, even when processing fully visible faces. Additionally, children with more experience with people wearing masks and fewer contacts without facial masks (i.e., more COVID-19-related experiences) might show less accurate emotion labeling than children with less COVID-19-related experiences.

Second, because children's emotion recognition depends on the specific emotions (Gagnon et al., 2014; Guarnera et al., 2015) and some emotions are better recognized from certain facial parts than others, preschoolers' emotion labeling of fully visible faces after the COVID-19-related changes might depend on the specific emotion they see.

## 2. Methods

We preregistered the study (https://osf.io/qaxp7) and made the data collected in the present study and codes available on the Open Science Framework (OSF, https://osf.io/tmj2c/).

### 2.1. Participants

The data collected before the COVID-19 pandemic (no-COVID-19-experience sample) were taken from studies by Streubel et al. (2020) (Child Faces Task) and Widen and Russell (2010) (Adult Faces Task) with the kind permission of the authors^1^. The sample by Streubel et al. (2020) was collected in Germany in 2019 and consisted of 30 children (11 girls, 19 boys) at the age of 4.54–5.59 years (*M* = 5.05 years, *SD* = 0.33 years). In this sample, 71% children had at least one parent with a college degree. The sample by Widen and Russell (2010) was collected in the United States before 2010 and consisted of 40 children (20 girls, 20 boys) at the age of 4.00–5.75 years (*M* = 4.91 years, *SD* = 0.46 years). In this sample, parents' mean education level was a master's degree.

The final with-COVID-19-experience sample consisted of 99 children (49 girls, 50 boys) at the age of 4.50–5.50 years (*M* = 5.01 years, *SD* = 0.27 years). All children had a normal birth weight (>2,500 g), were born full term (37–42 weeks gestation), and had no diagnosed developmental disorders as reported by the parents. The sample included 51 monolingual and 48 bilingual children. The mean of parents' highest level of education was some form of higher education (e.g., higher technical college) with 77% of children having at least one parent with a university degree (either bachelor's or master's degree). Additional eight children participated but were excluded from all tasks for different reasons. One girl had to be excluded because of her limited language skills to understand the questions and stories. One girl did not give understandable answers to the questions and her data could therefore not be coded. With one boy the tasks could not be performed because of difficulties with calibrating the eye tracker. Two girls did not provide any answers and could therefore not be coded. Three girls had to be excluded because of technical problems. The participants were recruited through the database of the research unit Developmental Psychology: Infancy and Childhood of the University of Zurich. The database consists of children whose parents are interested in participating in studies and therefore signed up at an earlier point in time. Each child received a certificate and a small present (value ~5$) for their participation. Parents gave written informed consent. The ethics commission of the UZH Faculty of Arts and Social Sciences had approved the general procedure. All procedures were performed in accordance with the ethical standards of the 1964 Helsinki declaration and its later amendments. The data collection took place between July and November 2021. At this point in time, families had experienced two lockdowns (March–May 2020 and December–February 2021) with schools and kindergartens remaining closed during the first lockdown. From July 2020 to February 2022, a mask obligation for all public indoor places (including public transportation) had been established for people aged 12 years and older.

### 2.2. Instruments

#### 2.2.1. Facial emotion recognition

Both tasks were presented on a 17” (800, 600 px) computer screen. The Child Faces Task [adapted from the *Intelligence and Development Scales* (IDS); Grob et al., 2009] consisted of a set of 10 pictures of children showing one of five different emotional facial expressions (happiness, anger, fear, surprise, sadness; each emotion depicted twice by two different children). In line with the original task (Grob et al., 2009), the pictures were presented in a fixed order, beginning with the picture of the emotion happiness. The children were asked to indicate the emotion of the child in the picture by verbally labeling it. There was no time limit. If the child's answer was not specific enough (e.g., the child said the child on the picture was feeling “good” or “bad”), the experimenter asked the child to specify the answer. If the child described an appearance or behavior, the experimenter asked the child to name the emotion in this specific appearance or behavior. If the child named more than one emotion for a picture, the experimenter asked the child to choose the most fitting one. In order to categorize the answers as correct (score 1) or incorrect (score 0) in each trial, we used the scoring key of the original test in Standard German. We categorized children's answers as incorrect, if they described the positive or negative valence of the emotions (“good,” “bad”), if they gave no answer, or if they said that they did not know the answer. The dependent variable for the Child Faces Task was children's score of zero or one, considered separately for each trial^2^.

The Adult Faces Task (replication of Widen and Russell, 2010) included nine pictures of adults showing different emotional facial expressions (happiness, anger, fear, surprise, disgust, contempt, shame, embarrassment, and compassion; pictures originally from Haidt and Keltner, 1999). The task started with the emotion happiness, which was followed by the other emotions in random order. Before the task, a priming was performed to ensure that the target emotion labels were accessible to the children. The experimenter introduced each of the target emotion labels by asking the children, whether they sometimes encounter the different emotions (“What about angry? Do you sometimes feel angry?”). Then the experimenter led the children through the pictures by telling a story about a woman. The children were asked to verbally label the emotions shown in the pictures. There was no time limit and children's answers were not corrected. If they gave no answer, the experimenter tried different prompts (i.e., repeating the question, or asking the child to look closely; see Widen and Russell, 2010). If the child still did not respond, the experimenter moved on to the next emotional expression. After presenting all emotions, the experimenter returned to any expression to which the child had not responded. The experimenter did not use the word “emotion” at any time, provide any emotion labels, or otherwise instruct the child to use an emotion label, other than asking how the woman was feeling. We categorized children's answers as in the Child Faces Task (see above) but using the scoring key from the original study developed by Widen and Russell (2003). The dependent variable for the Adult Faces Task was children's score of zero or one, considered separately for each trial.

#### 2.2.2. Emotion-specific vocabulary

To control for emotion-specific vocabulary, we used an adapted version of the *Children's Emotion Vocabulary Vignettes Test* (CEVVT) by Streubel et al. (2020). We only used a selection of the original 20 vignettes, testing for the six basic emotions (joy, anger, disgust, sadness, surprise, and fear) and four secondary emotions (guilt, shame, envy, and pride) in 10 vignettes. The selection was based on variance in response rate in the original sample (Streubel et al., 2020) at our target age of 4.5–5.5 years. Furthermore, we translated the selected vignettes of the original CEVVT from Standard German to Swiss German and ran a prestudy with adults for validation^3^.

The 10 vignettes showed a child in a typical emotion-provoking situation with emotion-specific facial and bodily expressions, physiological reactions, and thoughts. Each vignette comprised a picture and an audio-recorded gender-matched text that was presented simultaneously. The pictures and audio recordings were presented on a Microsoft Yoga laptop with a 14” touch-screen display using PowerPoint. Children were asked a comprehension question about the vignette itself and prompted to indicate how the child in the vignette was feeling. For further details on the randomization and procedure of this task see the original paper by Streubel et al. (2020). To categorize children's answers as correct (score 1) or incorrect (score 0), we used a self-developed scoring key based on the original study (Streubel et al., 2020), the scoring key of the IDS (Grob et al., 2009), and the answers of the adults in the prestudy. The measure was used to assess how many of the labels needed for the Child Faces Task the children actually produced. The dependent variable was a score ranging from 0 to 5, counting the number of correctly labeled emotions that were also used in the Child Faces Task (happiness, anger, fear, surprise, sadness).

#### 2.2.3. COVID-19-related-experiences questionnaire

We measured children's experience with pandemic-related changes in social interactions *via* a self-developed parental questionnaire. The questionnaire was filled out on a tablet by the parent accompanying the child during study participation. As a measure of exposure to facial masks, we assessed the number of hours per week children spent with adults wearing a facial mask (*mask exposure*, see Figure A1 for the distribution of parents' answers). To help caregivers with estimating the time their children spent with adults wearing masks, we asked separately about different places and situations (e.g., time spent in Kindergarten; see Appendix). For our analyses, we created a sum score across all these places/situations. As a measure of how many people children saw without facial mask we assessed the number of contacts per week children had with adults not wearing a facial mask with a single question (*without-mask contacts*, see Appendix for questionnaire).

#### 2.2.4. Gaze behavior

We measured children's gaze behavior during the emotion labeling tasks with an eye-tracking system (Eyelink 1000Plus, SR Research, sample rate: 500 Hz). A five-point calibration with an animated target was performed. After every three trials, a drift check and, if necessary (deviation >1° visual degrees), a re-calibration were performed. We analyzed children's fixation duration to the eyes and the mouth of the person on the picture presented. Fixations were defined using the default parameters of EyeLink 1000Plus (Data Viewer software). For each data sample, a parser computes instantaneous velocity and acceleration and compares these to velocity and acceleration thresholds. Under default settings, saccade onset (fixation offset) is signaled when either velocity or acceleration go above thresholds of 30 °/*s* and 8, 000 °/s^2^ respectively, and the eye has traveled at least 0.1°. To further analyse children's fixations, we drew areas-of-interest (AOI) around the mouth and the eye area of the person shown in each picture. The mouth area was drawn to resemble a facial mask in size and form. The eye area was drawn to match the mouth area in size (in pixel). We analyzed children's fixation duration (in ms) to these two AOIs by calculating an eyes-to-mouth index (fixation duration to the eyes/eyes + mouth AOIs) for each picture and participating child. This normalization accounting for differences in overall looking behavior (for details see Supplementary material) allowed us to include the fixation behavior of all children in all trials in our analyses (i.e., there was no threshold of minimum overall looking behavior per trial for a child to be included in the analyses).

#### 2.2.5. Other measures

We assessed children's vocabulary in their mother tongues with the BILEX (for details, see Gampe et al., 2018), a touch-screen based vocabulary test. Children's Theory of Mind was measured *via* a parental questionnaire with the *Children's Social Understanding Scale* (CSUS; Tahiroglu et al., 2014). We also asked parents for demographic information on the number and order of siblings, birth year of the siblings, day-care hours, and parental education as an approximation of the socioeconomic status (SES). Furthermore, this procedure included two additional eye-tracking tasks (administered before the Adult and Child Faces Tasks) and one interactive task on gaze following behavior (administered after the Adult and Child Faces Tasks) for another study. These measures were not analyzed for this paper.

### 2.3. Procedure

All children were tested individually with at least one parent present. During the testing session, the experimenter and parents wore facial masks. For approximately 15 min, each child and their parent were in a reception room where the experimenter described the test procedure to the parent and handed them the consent form to sign. The experimenter played with the child until they seemed comfortable. The experimenter then asked child and parent to move to the laboratory.

The laboratory was unfurnished except for the test equipment. The children were seated in a highchair which was placed in front of a table or the eye tracker, depending on the task. The parents were always seated on a chair behind the child and were asked to fill out the questionnaire. One test session lasted up to 75 min. To keep children motivated throughout the whole study, we included a cover story about a treasure hunt at the end of which the children could select small gifts. To be in line with Streubel et al. (2020), we decided to use a fixed order for the tasks by introducing the CEVVT before the Child Faces Task (Grob et al., 2009) followed by the Adult Faces Task.

## 3. Results

### 3.1. Between-group analyses

To analyse the impact of pandemic-related changes in social interactions on children's emotion recognition, we ran three mixed models on children's labeling behavior in the Child Faces and the Adult Faces Task. The first two models included emotion (Child Faces Task: 5; Adult Faces Task: 9; with reference category “happiness”), group (no/with-COVID-19-experience; with reference category “no-COVID-19-experience”), their interaction, and age in months as fixed effects and participant as random effect. The third model was an additional model for the Child Faces Task, which also included children's emotion-specific vocabulary as a factor^4^.

#### 3.1.1. Child faces task

**Figure 1:**
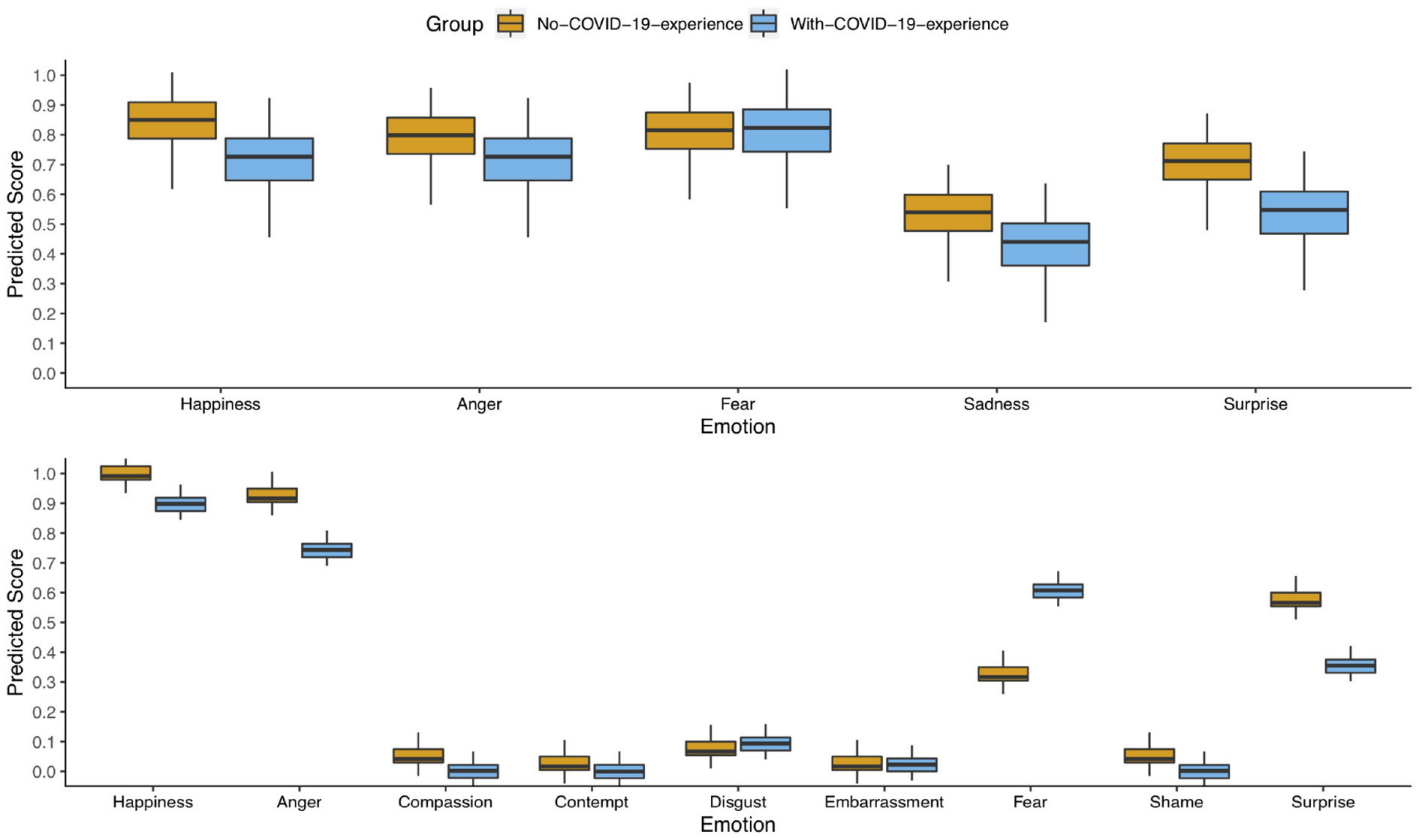
Children's predicted labeling score based on the according models in the Child Faces Task **(top)** and the Adult Faces Task **(bottom)** for the no-COVID-19-experience samples (orange) and the with-COVID-19-experience sample (blue). The higher the score, the more accurately children labeled the emotions depicted in the faces.

The results of the Child Faces Task showed a significant effect of the emotion sadness (*Estimate* = −0.310, *SE* = 0.080, *p* < 0.001) in such that sadness was recognized less accurately than happiness in child faces. Furthermore, older children labeled the emotions more accurately than younger children (*Estimate* = 0.012, *SE* = 0.005, *p* = 0.018). No other significant effects were found (see Table 1). Therefore, no significant difference between the two groups (no/with-COVID-19-experience sample) emerged in the Child Faces Task (*Estimate* = −0.118, *SE* = 0.070, *p* = 0.095, see Figure 1).

**Table 1:** Child faces task: association with pandemic-related changes in social interactions.

**Variables**	***Estimate***	***SE***	***df***	***t***	***p***
Intercept	0.121	0.308	130.900	0.391	0.696
Anger	−0.052	0.080	1135.000	−0.647	0.518
Fear	−0.034	0.080	1135.000	−0.431	0.666
Sadness	−0.310	0.080	1135.000	−3.881	< 0.001
Surprise	−0.014	0.080	1135.000	−1.725	0.085
Group	−0.118	0.070	782.500	−1.670	0.095
Age	0.012	0.005	124.000	2.399	0.018
Anger * Group	0.052	0.091	1135.000	0.568	0.570
Fear * Group	0.131	0.091	1135.000	1.444	0.149
Sadness * Group	0.025	0.091	1135.000	0.271	0.787
Surprise * Group	−0.041	0.091	1135.000	−0.446	0.655

The reference category for emotion was happiness and for group the no-COVID-19-experience sample.

In line with the first model, the results of the model including children's emotion-specific vocabulary revealed a significant effect of the emotion sadness (*Estimate* = −0.310, *SE* = 0.080, *p* < 0.001), age (*Estimate* = 0.010, *SE* = 0.005, *p* = 0.036), and emotion-specific vocabulary (*Estimate* = 0.309, *SE* = 0.061, *p* < 0.001). The more labels of the Child Faces Task children produced in the CEVVT, the more accurate their emotion labeling was. No other significant effects emerged (see Table A1 in Appendix).

#### 3.1.2. Adult faces task

Except for the emotion of anger, children labeled all emotions less accurately than the emotion happiness (see Table 2). Furthermore, older children were more accurate in labeling the emotions than younger children (*Estimate* = 0.005, *SE* = 0.002, *p* = 0.036). The model also revealed a significant interaction of the emotion fear and group (*Estimate* = 0.384, *SE* = 0.080, *p* < 0.001). Children in the with-COVID-19-experience sample labeled the fearful face more accurately than children in the no-COVID-19-experience sample. No other significant effects were found (see Table 2 and Figure 1).

**Table 2:** Adult faces task: association with pandemic-related changes in social interactions.

**Variables**	***Estimate***	***SE***	***df***	***t***	***p***
Intercept	0.709	0.146	163.000	4.855	< 0.001
Anger	−0.075	0.067	1080.000	−1.118	0.264
Compassion	−0.950	0.067	1080.000	−14.157	< 0.001
Contempt	−0.975	0.067	1080.000	−14.529	< 0.001
Disgust	−0.925	0.067	1080.000	−13.784	< 0.001
Embarrassment	−0.975	0.067	1080.000	−14.529	< 0.001
Fear	−0.675	0.067	1080.000	−10.059	< 0.001
Shame	−0.950	0.067	1080.000	−14.157	< 0.001
Surprise	−0.425	0.067	1080.000	−6.333	< 0.001
Group	−0.109	0.057	1207.000	−1.905	0.057
Age	0.005	0.002	134.000	2.114	0.036
Anger * Group	−0.080	0.080	1080.000	−0.999	0.318
Compassion * Group	0.053	0.080	1081.000	0.665	0.506
Contempt * Group	0.078	0.080	1081.000	0.980	0.328
Disgust * Group	0.120	0.080	1080.000	1.506	0.133
Embarrassment * Group	0.100	0.080	1081.000	1.243	0.214
Fear * Group	0.384	0.080	1081.000	4.804	< 0.001
Shame * Group	0.053	0.080	1081.000	0.665	0.506
Surprise * Group	−0.118	0.080	1081.000	−1.482	0.139

The reference category for emotion was happiness and for group the no-COVID-19-experience sample.

### 3.2. Within COVID-19-experience sample analyses

#### 3.2.1. COVID-19-related experiences

To assess the association of children's COVID-19-related experiences and their labeling behavior, we ran two mixed models on children's score in the Child Faces and the Adult Faces Task respectively in the with-COVID-19-experience sample only. We included emotion, mask exposure or without-mask contacts respectively, and their interaction as fixed effects and participants as random effects.

##### 3.2.1.1. Child faces task

The model on the association of children's mask exposure with their labeling behavior revealed a significant effect of the emotion sadness (*Estimate* = 0.204, *SE* = 0.094, *p* = 0.030). Sadness was labeled less accurately than happiness. No other significant effects were found (see Table A2 in Appendix). Similarly, the model including without-mask contacts showed a significant effect of the emotion sadness (*Estimate* = 0.288, *SE* = 0.084, *p* < 0.001) but no other significant effects (see Table A3 in Appendix). Sadness was labeled less accurately than happiness.

##### 3.2.1.2. Adult faces task

Similar to the between-group analyses, the models including mask exposure or without-mask contacts revealed that children labeled all emotions except for anger less accurately than happiness (see Tables A4, A5 in Appendix). No other significant effects were found.

#### 3.2.2. Gaze behavior

To explore the association of children's gaze behavior with their labeling and their COVID-19-related experiences, we ran two mixed-linear model on children's eyes-to-mouth index in the Child Faces and the Adult Faces Task. We included emotion, children's labeling behavior, their mask exposure, and without-mask contacts as fixed effects and participants as random effects.

**Figure 2:**
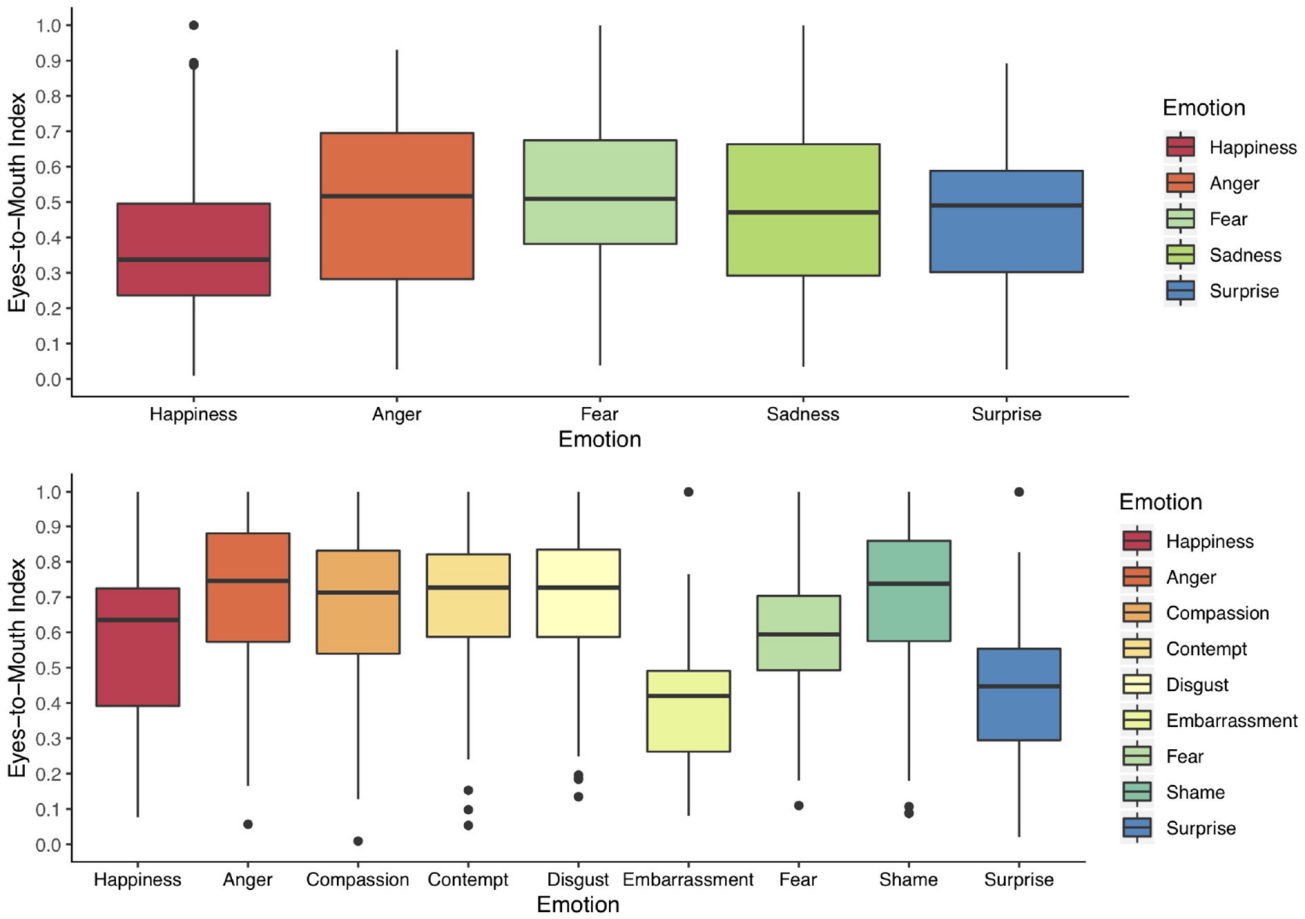
Children's eyes-to-mouth index for each emotion in the Child Faces Task **(top)** and the Adult Faces Task **(bottom)**. The greater the eyes-to-mouth index the longer children looked at the eye area compared to the mouth area. An eyes-to-mouth index of 0.5 indicates equivalent fixation duration to the eyes and the mouth area.

In the Child Faces Task, children had a greater eyes-to-mouth index, that is, looked longer to the eye area, in all emotions compared to the emotion happiness (see Figure 2 and Table A6 in Appendix). There was no significant effect of children's labeling behavior, *Estimate* = 0.002, *SE* = 0.011, *p* = 0.810, mask exposure, *Estimate* = 0.001, *SE* = 0.001, *p* = 0.599, or without-mask contacts, *Estimate* = −0.004, *SE* = 0.003, *p* = 0.222.

In the Adult Faces Task, the model revealed significant effects for all emotions except for fear (see Figure 2). For the emotions anger, compassion, contempt, disgust, and shame children had a greater eyes-to-mouth index, looked longer to the eye area, than for the emotion happiness. In contrast, children's eyes-to-mouth index for the emotions embarrassment and surprise was lower than for the emotion happiness (see Table A7 in Appendix). There was no effect of children's labeling behavior, *Estimate* = −0.004, *SE* = 0.019, *p* = 0.842, mask exposure, *Estimate* = −0.001, *SE* = 0.001, *p* = 0.230, or without-mask contacts, *Estimate* = −0.003, *SE* = 0.003, *p* = 0.326.

## 4. Discussion

The COVID-19 pandemic has changed people's social life. Children have increasingly interacted with adults wearing facial masks, seen fewer adults without facial masks, and probably encountered certain emotions in different frequencies than before the pandemic (e.g., more negative emotions). In this study, we explored whether these experiences are associated with children's emotion recognition. To address this question, we asked children to label emotions depicted in child and adult faces and assessed their gaze behavior. We compared data from other studies that assessed emotion recognition before the pandemic to data of other children measured in our own lab during the pandemic. In addition, we tested for potential associations with COVID-19-related experiences within the sample assessed during the pandemic.

Overall and in line with previous work on preschoolers (Gori et al., 2021; Schneider et al., 2021), the results of our study indicate no evidence for pandemic-related differences in social interactions in children's emotion labeling. We assume that children still received enough and enough variable input of non-masked faces to support their normal development of emotion recognition. This input may have come from their home environment (i.e., parents, siblings) or their peers. In (country, blinded), where the study was conducted, preschoolers were never obliged to wear masks, only their teachers were. Since emotion recognition from child and adult faces does not differ (Hall et al., 1999; Guyer et al., 2007), children's performance may have benefitted not only in the Child Faces Task but also in the Adult Faces Task from the unchanged facial input from their peers. In addition, the children participating in the current study were already at preschool age. Therefore, they had 3–4 years of experience with non-masked faces before the beginning of the pandemic. While children's performance was still not at ceiling, their previous years of normal facial input may have contributed to the current findings.

### 4.1. Comparing children with and without COVID-19 pandemic-related experiences

Across the samples assessed before and during the pandemic, there were some differences in how accurately the different emotions were labeled. In line with previous research (Boyatzis et al., 1993; Widen and Russell, 2008), some emotions (e.g., happiness) were recognized more accurately from both child and adult faces than others (e.g., sadness). Furthermore, in accordance with an ongoing development of emotion recognition in preschool years (Widen and Russell, 2003; Herba et al., 2006), the analyses revealed a significant effect of age. Independent of the sample, older children more often labeled the emotions shown in child and adult faces accurately than younger children.

In the Child Faces Task, children who knew more emotion labels were more accurate in recognizing emotions. This speaks to an influence of emotion label knowledge on children's performance. That is, the task cannot distinguish between children who do not recognize the emotion and children who do not know the respective emotion word. Therefore, measuring children's emotion recognition *via* labeling behavior may result in a biased picture in such that emotion recognition of children with a low emotion-specific vocabulary is underestimated.

Similar to the Child Faces Task, no significant effect of no/with-COVID-19-experience sample emerged in the Adult Faces Task. However, we found a significant interaction of group and fear in such that children during the pandemic recognized fear better than children before the pandemic. Children may have experienced more fearful adult faces in the two pandemic years than before (Ayenigbara et al., 2020; Chee, 2020; de Leo and Trabucchi, 2020). Especially their parents are likely to have shown more concern, anxiety, and depressive symptoms (Russell et al., 2020; Cerniglia et al., 2021). Staying at home during lockdowns has posed a strain on families. Many parents worked in home office while taking care of their children. In combination with lower social support this led to increased stress levels, and exhaustion (Lannen et al., 2020). The increased input of negatively valenced and especially fearful faces could have resulted in children's more accurate emotion recognition. Supporting this, the effect was specific to adult faces and we found no interaction of group and the emotion fear in the Child Faces Task. Alternatively, in line with previous work (Kestenbaum, 1992; Gagnon et al., 2014; Kim et al., 2022), children may have focused mostly on the eyes when labeling fearful faces. Since the eyes remain visible even when masks are worn, the increased input of masked faces might have supported children's recognition of fear in faces. Accordingly, our eye-tracking results do show a focus on the eyes, similar to recent findings in adults (Barrick et al., 2021). However, this effect was not specific to fear and children looked longer to the eyes than the mouth for most of the emotions. Furthermore, because gaze behavior was not recorded in the two studies that provided the data for the no-COVID-19-experience sample, it was not possible to compare children's gaze behavior in the Adult Faces Task to before the pandemic. In sum, based on our data we cannot draw a definite conclusion on the reason for children's increased recognition of fearful faces during the pandemic.

### 4.2. Associations between pandemic-related experiences, emotion labeling, and gaze behavior

Equivalent to our between-group analyses, the analyses within the COVID-19-experience sample showed no significant association of our measures of pandemic-related changes in social interactions (mask exposure, without-mask contacts) with children's emotion labeling. In accordance with previous studies (Kestenbaum, 1992; Guarnera et al., 2017), we found that children's gaze behavior differed between emotions. For most emotions, children seemed to look longer to the eyes than the mouth, while the reverse pattern emerged for emotions such as embarrassment or surprise. There was no significant association of children's labeling behavior and their gaze behavior. In contrast to previous work (Kestenbaum, 1992; Gagnon et al., 2014; Guarnera et al., 2015), our data therefore suggest that there is no “optimal” looking pattern, which is related to a better emotion recognition.

### 4.3. Limitations

As mentioned before, measuring emotion recognition *via* labeling behavior has its pitfalls and relies on children's emotion-specific vocabulary. Furthermore, especially the stimuli used in the Adult Faces Task may not have captured children's true emotion recognition. The pictures were more than 10 years old, black-and-white, and emotions were acted out in an exaggerated way. This contrasts children's everyday experiences with emotions. There, children encounter and read emotions of different intensities based on multimodal cues, which include facial features but also the tone of voice or body posture (Meeren et al., 2005; Aviezer et al., 2008). Therefore, the stimuli (i.e., static pictures) may not have measured children's every day ability to recognize emotions. Also, the number of items in these emotion recognition tasks is quite limited so that the response to each individual item has a relatively strong influence on the overall score. However, since this study is based on a “natural intervention,” we had to rely on data assessed before the pandemic that was available and accessible. Consequently, we had to use the measures of emotion recognition employed in previous studies. For the same reason, we were also not able to compare children's gaze behavior during and before the pandemic (i.e., there was no behavioral data available that included gaze behavior). This additional data would have allowed analysing whether explicit (i.e., labeling behavior) and implicit (i.e., gaze behavior) measures converge or whether they measure different behaviors and processes. Furthermore, any differences between the samples in our study may not have been due to pandemic-related but cultural reasons. While the sample for the Child Faces Task was assessed right before the pandemic in Germany, a culture very similar to (country, blinded), the children in the Adult Faces Task were from the United States and their emotion labeling was measured before 2010. However, we are not aware of any differences between the cultures in (country, blinded) and the United States that influence children's emotion labeling. Additionally, the fact that our findings are largely consistent between the Child Faces and the Adult Faces Task speaks toward their robustness and validity. Finally, while our study suggests no significant short-term effects of pandemic-related changes in social interactions on children's emotion labeling, it does not rule out any long-term influences that occur later in children's development. This should be the target of future research.

### 4.4. Conclusion

In sum, our study indicates that the COVID-19 pandemic and the according changes in social interactions such as meeting fewer people, or seeing more people wearing masks, do not substantially relate to preschoolers' emotion labeling. Preschoolers likely have received enough input of non-masked faces to support their normal development of emotion recognition.

## Data availability statement

The datasets presented in this study can be found in online repositories. The names of the repository/repositories and accession number(s) can be found at: https://osf.io/tmj2c/.

## Ethics statement

The studies involving human participants were reviewed and approved by UZH Faculty of Arts and Social Sciences. Written informed consent to participate in this study was provided by the participants' legal guardian/next of kin.

## Author contributions

SW and LM: conceptualization, data analyses, supervision of SA, and writing, review and editing the paper. SA: conceptualization, data collection and coding, some data analyses, and writing and editing the paper. MD: conceptualization and writing, review, and editing the paper. All authors contributed to the article and approved the submitted version.

## Funding

This paper has been funded by the Foundation for Research in Science and the Humanities at the University of Zurich (https://www.research.uzh.ch/en/funding/researchers/stwf.html, grant number: STWF-21-018) with CHF 20'860.

## Conflict of interest

The authors declare that the research was conducted in the absence of any commercial or financial relationships that could be construed as a potential conflict of interest.

## Publisher's note

All claims expressed in this article are solely those of the authors and do not necessarily represent those of their affiliated organizations, or those of the publisher, the editors and the reviewers. Any product that may be evaluated in this article, or claim that may be made by its manufacturer, is not guaranteed or endorsed by the publisher.

## Footnotes

^1^In the following, we report parents highest level of education as an approximation of children's socioeconomic status (SES). However, because the no/with-COVID-19-experience samples were collected in different countries and non-comparable education systems, we only report SES descriptively.

^2^We pre-registered that children would be excluded if they had one missing label of the five emotions. However, the majority of children did not provide answers to all emotions. To avoid major data loss, we decided to include data from children who had missing labels.

^3^The results of this prestudy are summarized in the Supplementary material and available on the OSF https://osf.io/tmj2c/.

^4^All variance inflation factors (VIFs) of the linear predictors of the models were around 1, indicating no multicollinarity.
