# Supplementary material for: Exploring the role of COVID-19 pandemic-related changes in social interactions on preschoolers' emotion labeling
Source: Front Psychol. 2022 Sep 28;13:942535. doi: 10.3389/fpsyg.2022.942535 (PMC9554629; doi:10.3389/fpsyg.2022.942535)
Supplement: Supplementary file 1 [file Data_Sheet_1.pdf]

Supplemental Material

Supplemental material of the paper "Exploring the Role of COVID-19 Pandemic-Related Changes in Social Interactions on Preschoolers' Emotion Labelling" authored by (authors, blinded for review purposes)

Prestudy for CEVVT Items

We translated selected vignettes (10 vignettes; joy, anger, disgust, sadness, surprise, fear, guilt, shame, envy, and pride) of the original *Children's Emotion Vocabulary Vignettes Test* (CEVVT) from Standard-German to (country language, blinded for review). To validate the new language-version, we conducted an online prestudy with adults.

The sample of this prestudy consisted of 40 participants (34 female, 6 male) at the age of 19 to 47 years (M = 35.97 years, SD = 6.86 years). All participants were bilingual Standard German - Swiss German, either speaking both languages as their mother tongue or having lived in either country for at least 10 years. Participants received credits for their psychology degree after their participation.

We asked the adults to label the emotion expressed in each vignette and rate the intensity of the emotion on a scale from "not at all strong" (0) to "very strong" (100) using a slide controller. We created two counterbalanced conditions in such that each participant saw half of the vignettes in Swiss German and the other half in Standard German. The vignettes were presented in the same order to all participants.

We analysed whether the intensity of the emotions expressed in the vignettes was rated as equally strong in both languages. The results showed a significant difference for the emotions envy (p = .003) and shame (p = .049). In comparison to the Swiss German vignettes, the emotions expressed in the Standard German vignettes were rated as being lower in intensity. For all other emotions, results revealed no significant difference (see Figure 1). The results of adult's labelling behaviour can be found on the Open Science Framework (https://osf.io/tmj2c/).

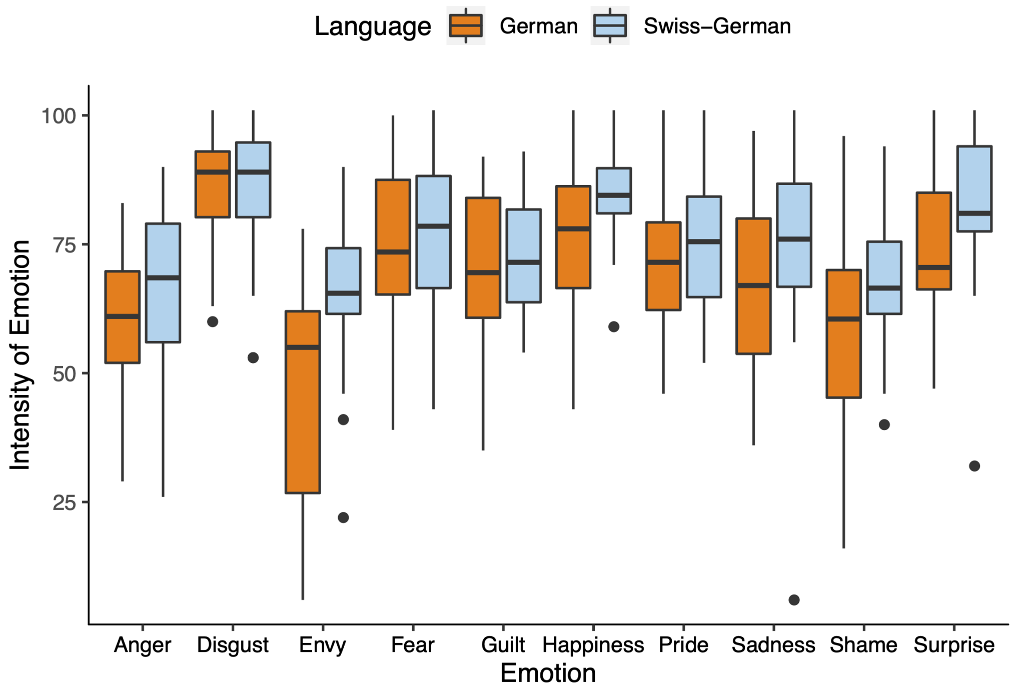

Figure 1

Intensity of the emotion expressed in each of the 10 vignettes in the Standard German (orange) and the Swiss German (blue) version.

Emotion Labelling

To provide more information on children's labelling behaviour, we report the mean percentage of emotions labelled correctly per task. On average, in the Child Faces Task children in the no-COVID-19-experience sample labelled 92% ($SD = 11\%$) of emotions correctly (i.e., at least one of two pictures of an emotion were correctly labelled). In the with-COVID-19-experience sample, 87% ($SD = 18\%$) of emotions were labelled correctly. In the Adult Faces Task, children in the no-COVID-19-experience sample labelled 34% ($SD = 10\%$) and children in the with-COVID-19-experience sample labelled 30% ($SD = 12\%$) of emotions correctly. We additionally calculated the percentage of children correctly labelling the emotion in each picture. This data is shown in Figure 2 for the Child Faces Task and in Figure 3 for the Adult Faces Task.

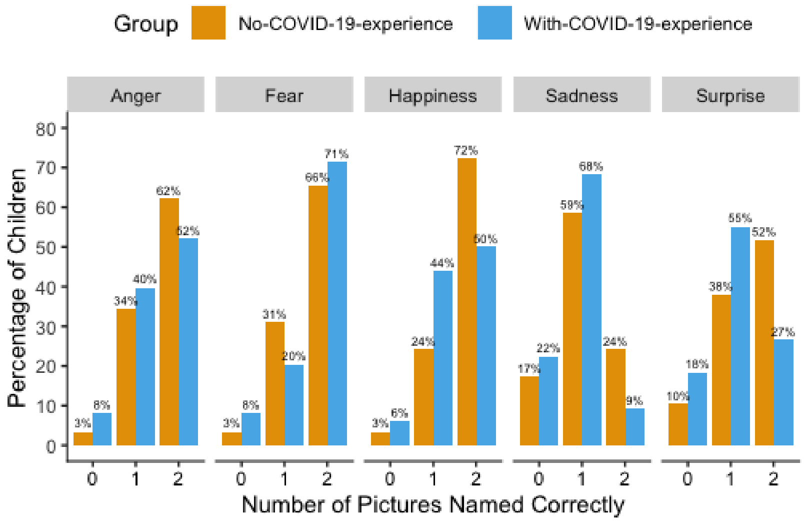

Figure 2

Percentage of children correctly labelling the emotion shown in each picture for the no-COVID-19-experience sample (orange) and the with-COVID-19-experience sample (blue) in the Child Faces Task. For each emotion, children saw two different pictures.

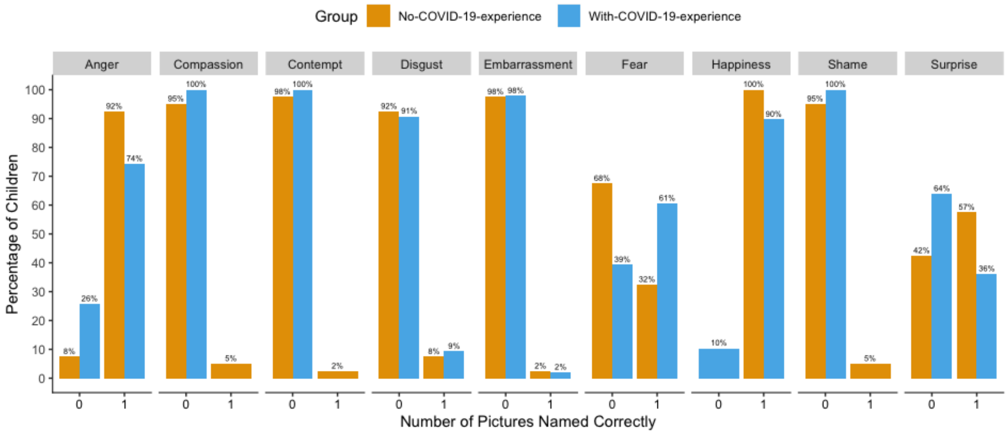

Figure 3

Percentage of children correctly labelling the emotion shown in each picture for the no-COVID-19-experience sample (orange) and the with-COVID-19-experience sample (blue) in the Adult Faces Task. For each emotion, children saw one picture.

Total Fixation Duration

We additionally provide information on children's total fixation duration per emotion for both tasks (see Table 1). Children's total fixation duration to the screen is likely to

be influenced by many factors such as children's individual attention span, the picture shown, or the duration of the trial, which was terminated when the child labelled the emotion. We normalised children's fixation data for our analyses in the paper by calculating the eyes-to-mouth index to account for these differences in children's total fixation duration.

Table 1

Children's Total Fixation Duration

Emotion	*M*	*SD*
	Child Faces Task	
Anger	8807	4698
Fear	12950	7728
Happiness	11905	5941
Sadness	10071	5432
Surprise	14278	7701
	Adult Faces Task	
Anger	4954	1946
Compassion	5964	3738
Contempt	7873	4695
Disgust	5410	2947
Embarrassment	5716	3840
Fear	5265	2550
Happiness	5097	2068
Shame	5510	3809
Surprise	5838	2624

Note. We report mean and standard deviation in milliseconds.
