# Supplementary material for: Exploring the role of COVID-19 pandemic-related changes in social interactions on preschoolers' emotion labeling
Source: Front Psychol. 2022 Sep 28;13:942535. doi: 10.3389/fpsyg.2022.942535 (PMC9554629; doi:10.3389/fpsyg.2022.942535)
Supplement: Supplementary file 2 [file Data_Sheet_2.pdf]

Appendix

Mask Exposure and People Without Mask Questionnaire

Please reflect upon the past month. In a normal week, how many hours does your child observe you or others wearing masks in the following activities?

Regular Activities

Kindergarten

- When did your child start attending kindergarten? If your child does not attend kindergarten, please leave this field empty.
- How many hours per week does your child attend kindergarten?
- Do the adults (e.g., caregivers) wear a face mask in kindergarten?

Out-of-home care

- Does your child receive care outside of immediate the family? (as example: institutional daycare, nanny, grandparents, day family, babysitter)
- How many hours a week does your child receive care outside of the immediate family?
- Do the people wear face masks when taking care of your child outside of the immediate family?

Club activities and courses

- Does your child regularly participate in club activities or attend classes (e.g., swimming lessons, music course)?
- How many hours per week does your child participate in club activities or attend classes?
- Do the instructors of the classes wear face masks?

Irregular Activities

1. Public

How many hours per week does your child see you or someone else wearing a mask in public? Please think about the last month. For example:

- Shopping for food, clothes, etc.
- Church service
- Visits to authorities and doctors
- Use of public transport
- In pedestrian zones or public places
- In a restaurant or café

2. Free time

How many hours per week does your child see you or someone else wearing a mask during free time activities? Please think about the last month. For example:

- Playground visit
- Leisure activities (e.g. museum, zoo)
- Sports or cultural events (e.g. football match, cinema, theater, concerts)
- Playing with other children

3. Visit

How many hours per week does your child see you or someone else with a mask during visits? Please think about the last month. For example:

- Visiting others at home
- Having a visit at home oneself

Contacts Without Mask

In an average week, how many adults without a mask does your child see for more than 10 minutes? Please indicate the number of adults. Think of household members and caregivers as well as relatives, friends, and acquaintances.

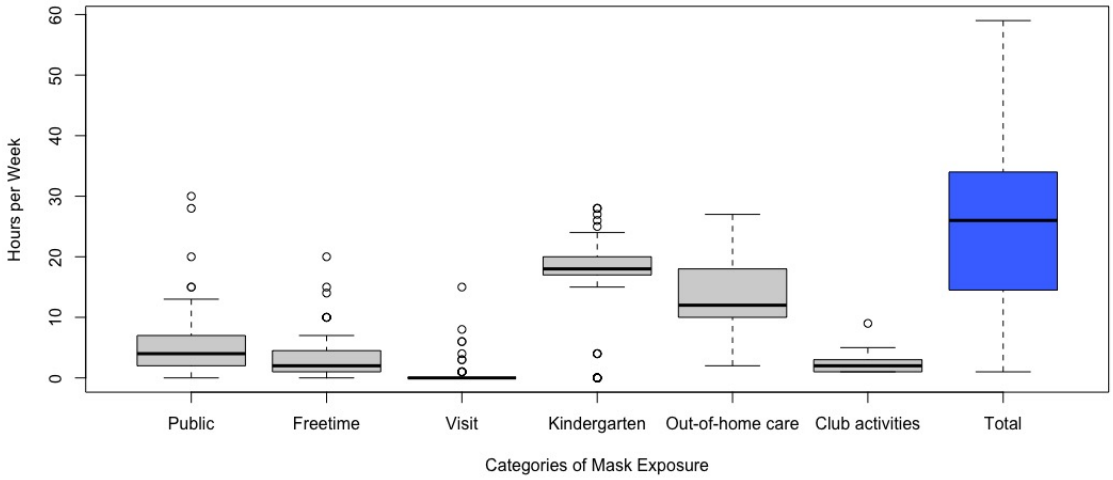

Figure A1

Hours per week children spent with people wearing masks in kindergarten, out-of-home care, club activities, public places, their free time and when visiting others or being visited. Furthermore, children saw $M = 9.17$ *(*$SD = 5.78$*) people without a mask per week for at least 10 minutes.*

Between-Group Analyses

Table A1

Child Faces Task: Association with Pandemic-Related Changes in Social Interactions and Children's Emotion-Specific Vocabulary

Variables	*Estimate*	*SE*	*df*	*t*	*p*
Intercept	0.123	0.283	130.200	0.436	.664
Anger	-0.052	0.080	1126.000	-0.646	.519
Fear	-0.034	0.080	1126.000	-0.430	.667
Sadness	-0.310	0.080	1126.000	-3.874	< .001
Surprise	-0.0138	0.080	1135.000	-1.725	.085
Group	-0.121	0.069	876.000	-1.769	.077
Age	0.010	0.005	122.000	2.117	.036
Emotion-Specific Vocabulary	0.309	0.061	122.000	5.045	< .001
Anger * Group	0.052	0.091	1126.000	0.567	.571
Fear * Group	0.132	0.091	1126.000	1.450	.147
Sadness * Group	0.032	0.091	1126.000	0.350	.726
Surprise * Group	-0.042	0.091	1126.000	-0.465	.642

Note. The reference category for emotion was happiness and for group no-COVID-19-experience sample.

Within COVID-19-Experience Sample Analyses

Table A2

Child Faces Task: Association with Children's Mask Exposure

Variables	*Estimate*	*SE*	*df*	*t*	*p*
Intercept	0.724	0.073	584.700	9.918	< .001
Anger	-0.034	0.094	874.000	-0.361	.718
Fear	0.078	0.094	874.000	0.836	.403
Sadness	-0.204	0.094	874.000	-2.175	.003
Surprise	-0.171	0.094	874.000	-1.821	.069
Mask Exposure	-0.000	0.002	584.700	-0.071	.944
Anger * Mask Exposure	0.001	0.003	874.000	0.409	.682
Fear * Mask Exposure	0.000	0.003	874.000	0.225	.822
Sadness * Mask Exposure	-0.003	0.003	874.000	-0.992	.322
Surprise * Mask Exposure	-0.003	0.003	874.000	-0.097	.923

Note. The reference category for emotion was happiness.

Table A3

Child Faces Task: Association with Children's Without-Mask Contacts

Variables	*Estimate*	*SE*	*df*	*t*	*p*
Intercept	0.656	0.065	581.500	10.071	$< .001$
Anger	0.003	0.084	865.000	0.032	.975
Fear	0.078	0.084	865.000	1.935	.053
Sadness	-0.288	0.084	865.000	-3.442	$< .001$
Surprise	-0.152	0.084	865.000	-1.816	.070
Without-Mask Contacts	0.007	0.006	581.500	1.092	.275
Anger * Without-Mask Contacts	0.000	0.008	865.000	0.035	.972
Fear * Without-Mask Contacts	-0.006	0.008	865.000	-0.829	.407
Sadness * Without-Mask Contacts	0.000	0.008	865.000	0.064	.949
Surprise * Without-Mask Contacts	-0.003	0.008	865.000	-0.327	.744

Note. The reference category for emotion was happiness.

Table A4

Adult Faces Task: Association with Children's Mask Exposure

Variables	*Estimate*	*SE*	*df*	*t*	*p*
Intercept	0.862	0.067	844.800	12.930	< .001
Anger	-0.123	0.092	760.000	-1.330	.184
Compassion	-0.861	0.092	760.700	-9.349	< .001
Contempt	-0.862	0.093	760.800	-9.299	< .001
Disgust	-0.681	0.091	762.400	-7.445	< .001
Embarrassment	-0.853	0.093	761.400	-9.176	< .001
Fear	-0.273	0.094	762.900	-2.921	.004
Shame	-0.861	0.092	761.300	-9.370	< .001
Surprise	-0.462	0.092	760.200	-5.003	< .001
Mask Exposure	0.001	0.002	844.800	0.602	.548
Anger * Mask Exposure	-0.001	0.003	760.000	-0.390	.696
Compassion * Mask Exposure	-0.001	0.003	761.900	-0.441	.660
Contempt * Mask Exposure	-0.001	0.003	762.200	-0.426	.670
Disgust * Mask Exposure	-0.005	0.003	761.800	-1.543	.123
Embarrassment * Mask Exposure	-0.000	0.003	760.800	-0.283	.777
Fear * Mask Exposure	-0.000	0.003	762.600	-0.223	.823
Shame * Mask Exposure	-0.001	0.003	760.700	-0.442	.659
Surprise * Mask Exposure	-0.003	0.003	760.500	-0.987	.324

Note. The reference category for emotion was happiness.

Table A5

Adult Faces Task: Association with Children's Without-Mask Contacts

Variables	*Estimate*	*SE*	*df*	*t*	*p*
Intercept	0.870	0.060	836.000	14.464	< .001
Anger	-0.164	0.083	752.000	-1.962	.050
Compassion	-0.870	0.083	753.600	-10.443	< .001
Contempt	-0.870	0.084	753.600	-10.415	< .001
Disgust	-0.764	0.083	752.600	-9.182	< .001
Embarrassment	-0.866	0.084	752.800	-10.356	< .001
Fear	-0.272	0.084	754.500	-3.233	< .001
Shame	-0.870	0.083	752.700	-10.470	< .001
Surprise	-0.467	0.083	753.600	-5.643	< .001
Without Mask Exposure	0.003	0.005	836.800	0.511	.610
Anger * Without-Mask Contacts	0.000	0.008	752.000	0.103	.918
Compassion * Without-Mask Contacts	-0.003	0.008	752.400	-0.368	.713
Contempt * Without-Mask Contacts	-0.003	0.008	752.400	-0.369	.712
Disgust * Without-Mask Contacts	-0.004	0.008	752.200	-0.553	.580
Embarrassment * Without-Mask Contacts	-0.000	0.008	752.200	-0.114	.909
Fear * Without-Mask Contacts	-0.001	0.008	753.600	-0.165	.869
Shame * Without-Mask Contacts	-0.003	0.008	752.200	-0.363	.717
Surprise * Without-Mask Contacts	-0.008	0.008	752.700	-1.008	.314

Note. The reference category for emotion was happiness.

Table A6

Child Faces Task: Association of Labelling Behaviour and COVID-19-Related Experiences with Eyes-to-Mouth Index

Variables	*Estimate*	*SE*	*df*	*t*	*p*
Intercept	0.376	0.059	97.120	6.323	< .001
Anger	0.131	0.014	813.600	9.322	< .001
Fear	0.154	0.014	813.300	10.894	< .001
Sadness	0.112	0.014	813.500	7.742	< .001
Surprise	0.096	0.014	813.300	6.779	< .001
Score	0.002	0.001	831.600	0.241	.810
Mask Exposure	0.001	0.001	89.830	0.528	.599
Without-Mask Contacts	-0.004	0.003	89.850	-1.229	.222

Note. The reference category for emotion was happiness.

Table A7

Adult Faces Task: Association of Labelling Behaviour and COVID-19-Related Experiences with Eyes-to-Mouth Index

Variables	*Estimate*	*SE*	*df*	*t*	*p*
Intercept	0.640	0.053	133.695	11.991	< .001
Anger	0.133	0.023	711.738	5.781	< .001
Compassion	0.087	0.028	721.836	3.055	.002
Contempt	0.103	0.029	722.598	3.585	< .001
Disgust	0.102	0.027	719.323	3.713	< .001
Embarrassment	-0.176	0.029	721.894	-6.121	< .001
Fear	0.020	0.024	713.630	0.818	.414
Shame	0.122	0.028	722.344	4.281	< .001
Surprise	-0.149	0.025	716.371	-5.870	< .001
Score	-0.004	0.019	738.990	-0.200	.842
Mask Exposure	-0.001	0.001	88.959	-1.209	.230
Without-Mask Contacts	-0.003	0.003	88.984	-0.987	.326

Note. The reference category for emotion was happiness.
